# Profound CD4+ T lymphocytopenia in human immunodeficiency virus negative individuals, improved with anti-human herpes virus treatment

**Published:** 2012-12-30

**Authors:** María Lilia Díaz Betancourt, Julio César Klínger Hernández, Victoria Eugenia Niño Castaño

**Affiliations:** a Immunology and Infectious Diseases Research Group, Department of Pathology, Faculty of Health Sciences, Universidad del Cauca, Colombia. E-mail: mdiaz1903@hotmail.com

**Keywords:** cytomegalovirus, epstein barr virus infection, idiopathic CD4-positive, lymphocytopenia etiology, herpesvirus 7, human, herpesvirus 6, acquired cellular immunodeficiency HIV negative

## Abstract

Lymphocytopenia and CD4+ T lymphocytopenia can be associated with many bacterial, fungal, parasite and viral infections. They can also be found in autoimmune and neoplastic diseases, common variable immunodeficiency syndrome, physical, psychological and traumatic stress, malnutrition and immunosuppressive therapy. Besides, they can also be brought into relation, without a known cause, with idiopathic CD4+ T lymphocytopenia. Among viral infections, the Retrovirus, specially the human immunodeficiency virus, is the most frequently cause. However, many acute viral infections, including cytomegalovirus and Epstein Barr virus can be associated with transient lymphocytopenia and CD4+ T lymphocytopenia. As is well known, transient lymphocytopenia and CD4+ T lymphocytopenia are temporary and overcome when the disease improves. Nonetheless, severe CD4+ T Lymphocytopenia associated with chronic infections by human herpes virus has not been reported. We describe 6 cases of human immunodeficiency virus negative patients, with chronic cytomegalovirus and Epstein Barr virus infections and profound lymphocytopenia with clinical symptoms of cellular immunodeficiency. These patients improved rapidly with ganciclovir or valganciclovir treatment. We claim here that it is important to consider the chronic human herpes virus infection in the differential diagnosis of profoundly CD4+ T lymphocytopenia etiology, when human immunodeficiency virus is absent, in order to start effective treatment and to determine, in future studies, the impact of chronic human herpes virus infection in human beings' health.

## Abstract (es)

Múltiples enfermedades bacterianas, micóticas, parasitarias y virales pueden asociarse con linfocitopenia y linfocitopenia CD4+. También enfermedades autoinmunes, neoplásicas, inmunodeficiencia común variable, estrés físico, sicológico o traumático, la malnutrición y el tratamiento con inmunosupresores. Esta condición también se presenta sin causa aparente y es conocida como linfocitopenia T CD4+ idiopática. Entre las infecciones virales, los retrovirus, especialmente el virus de inmunodeficiencia humana, es la más frecuente causa, pero muchas otras infecciones virales agudas, entre ellas, la mononucleosis por citomegalovirus y por Epstein Barr virus, se asocian con linfocitopenia total y linfocitopenia T CD4+, que son transitorias y se recuperan cuando la enfermedad mejora. Una linfocitopenia grave asociada con infección crónica por virus herpes humanos y que mejore con el tratamiento de ellos, no ha sido publicada. Se describen 6 pacientes, negativos para virus de inmunodeficiencia humana, con linfocitopenia total y linfocitopenia T CD4+ graves y con manifestaciones clínicas de inmunodeficiencia celular, quienes respondieron rápidamente al tratamiento con ganciclovir o valganciclovir. Es importante considerar la infección crónica por virus herpes humanos en el diagnóstico diferencial de la etiología de la linfocitopenia T CD4+ en individuos no infectados por el virus de inmunodeficiencia humana, para iniciar un tratamiento efectivo de los pacientes y determinar en futuros estudios el impacto de la infección crónica por herpes virus en la salud humana.

## Introduction

Lymphocytopenia is defined as a low lymphocytes count in peripheral blood, below 1,500 cells/*mm**^3^* in adults and below 3,000cells/*mm**^3^* in children. Transient lymphocytopenia and CD4+ T lymphocytopenia without clinical immunodeficiency have been seen in a variety of conditions such as bacterial, parasitic, fungal and viral infections. Other acute diseases such as traumas, sepsis and burns have also been seen. In addition to this, severe stress and prolonged or intense physical exercise are thought to have induced them. Other diseases associated with them are systemic lupus erythematosus (SLE), rheumatoid arthritis, Sjogren's syndrome, sarcoidosis and malnutrition. Also CD4+ T lymphocytopenia can be the result of corticosteroid or citotoxic treatments^1^.

Prolonged CD4+ T lymphocytopenia is found in human immunodeficiency virus (HIV) infection, other retroviruses, common variable immunodeficiency of adult onset and idiopathic CD4+ T lymphocytopenia (ICL)^1^.

In regard to virus infection and lymphocytopenia or CD4+ T lymphocytopenia, a great variety of acute infections are known to produce alterations such as the cold virus, hepatitis B, hepatitis C, influenza, dengue, herpes simplex, herpes virus 6, herpes virus 7, parvovirus B19, varicella zoster, acute cytomegalovirus (CMV) and Epstein Barr virus (EBV). Patients with mononucleosis by CMV had a decreased number of CD4 and a decreased CD4/CD8 ratio on average 0,2, while 10 healthy individuals had 73% more CD4 cells that CMV patients and CD4/CD8 ratio on average ^1^ ^,^ ^7^. Reviewed by Walker UA *et al*. 2006^1^. These abnormalities improved when the recovery of the acute illness occurred. Contrary to CD4+ T lymphocytopenia described for acute CMV mononucleosis, 31 CMV asymptomatic carriers showed a normal count of CD4+ T cells, on average 1,215 (685-2,200)/*mm**^3^*, without significant difference with 1,087 (522-1,776)/*mm**^3^* in 39 CMV seronegative individuals, as reviewed by Walker UA *et al*. 2006^1^. Profound CD4+ T lymphocytopenia has not been reported in chronic CMV infection, although it induces alterations in the composition of T cell subsets including an increase of specific CD4+ T and CD8+ T cells, which are more frequently CD45 RO+, CD28-, CD27- and CD57+^2^. These changes indicate an immune deviation toward memory phenotype and senescence that is more marked in elderly CMV seropositive individuals.We have seen various cases of profound CD4+ T lymphocytopenia with clinical immunodeficiency in HIV negative people, all of whom showed laboratory evidence of chronic CMV and EBV infections and active replication of CMV, evidenced by positive IFI for the PP65 antigen of CMV in peripheral blood. All of these patients recovered rapidly with ganciclovir or valganciclovir treatment. This report describes the first 6 illustrative cases. The recognition of this association not reported previously may be important for effective treatment of these patients and for opening new research horizons.

## Patients description

### Patient 1:

**Figure 1:**
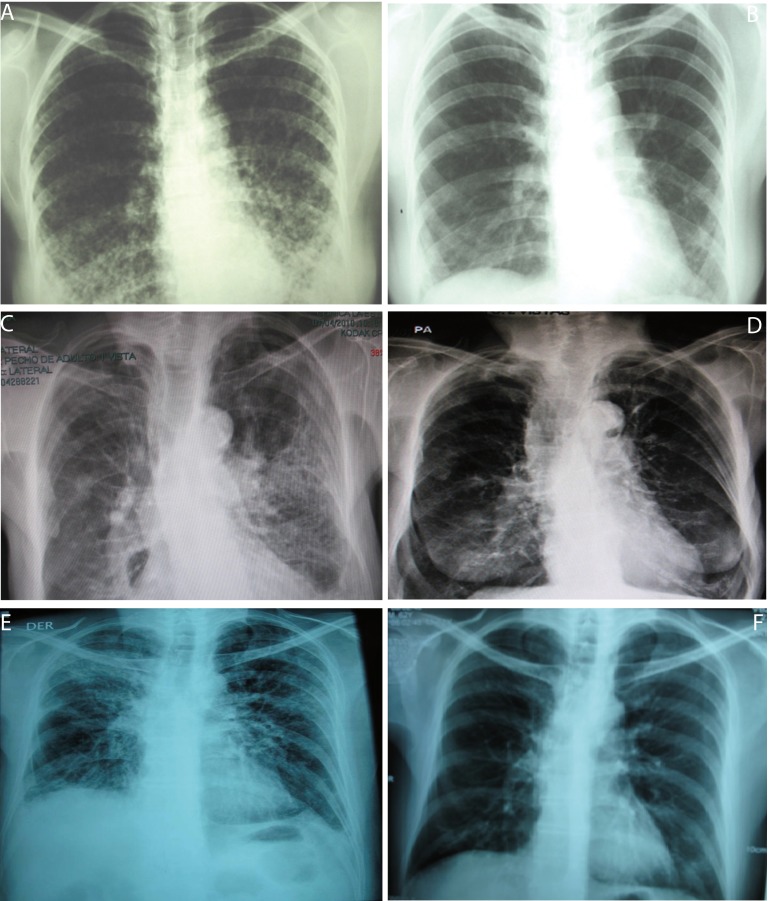
Chest X-ray of 3 patients. A and B: Chest X-ray of patient 1, December 2001 and July 2003 before and 22 months after ganciclovir treatment end, respectively. C and D: Chest X-ray of patient 5, April 2010 and September 2010, before and two months after ganciclovir-vaganciclovir treatment end, 4 months after trimethoprin sulfamethoxazole treatment end and 1 month after voriconazol treatment end, respectively. E and F: Chest X-ray of patient 6, November 2009 and February 2010, before and two months after trimethoprin sulfamethoxazole end and at ganciclovir treatment end, respectively.

**Figure 2.**
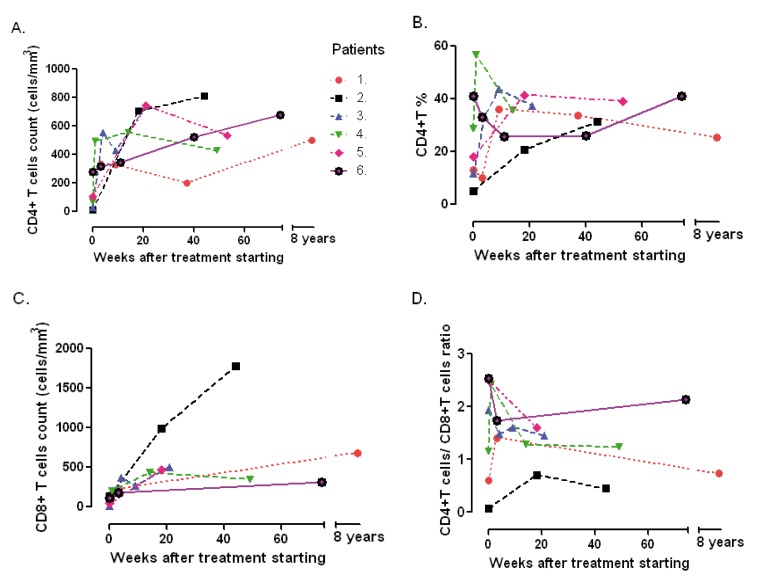
CD4+ T cells, percentage of them CD8+ T lymphocytes and CD4+/CD8 T lymphocytes ratio evolution during ganciclovir treatment. A panel: CD4+ T lymphocytes/mm3, B panel: % of CD4+ T lymphocytes, C panel: CD8+ T lymphocytes/mm3 and D panel: CD4+ T cells/CD8+ T cells ratio: Each point represents the basal value before the anti-human herpes virus treatment (the first point for each patient) and during the following weeks after it was started. A great increase of all basal parameters was observed in all six patients after ganciclovir or valganciclovir treatment.

A 38 year-old woman, heterosexual with no history of promiscuity, was admitted to a medical care hospital on January 21^th^, 2002 with a 3 month history of chills, fever and coughing with sputum production, progressive dyspnea, fatigue, weakness, anorexia and 10 kg weight loss. At the time of presentation she was being treated with 4 antituberculosis drugs for 34 days determined by bilateral reticulonodular interstitial infiltrates in her chest X-ray (Fig. 1A) and chest computed tomography (chest CT). Samples of bronchoalveolar lavage (BAL) were mycobacteria stain and culture negative. Transtracheal wash was fungus stain and culture negative too. She had a low level of hemoglobin (11.3 g/dL), hematocrit (37.7%), peripheral blood lymphocytes (699 /*mm**^3^*), CD4+ T cells/*mm**^3^* 92 (13.2%) and CD8+ T cells/*mm**^3^* 144 (Table 1 and Figure 2:A-C). Lung biopsy showed nonspecific focal, interstitial fibrosis, a few *Pneumocystis jiroveci (P. jiroveci)* and DNA of EBV by polymerase chain reaction (PCR). The patient's vital signs showed a temperature that ranged from 38 °C to 39.5 °C, respiratory rate of 40, pulse of 140, weight of 43 kg, height 1.62 m. Treatment with trimethoprin sulfamethoxazole and prednisone for 21 days was commenced on February 6^th^. The patient's temperature fell to 38 °C and pulse to 128, but she persisted tachypneic with a respiratory rate of 56 and coughing. Her weight decreased to 38 kg.

**Table 1:**
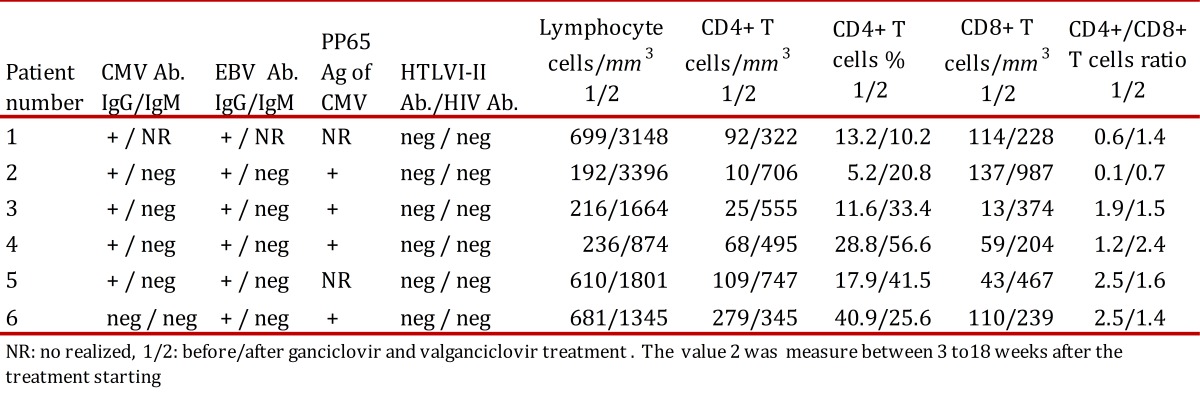
Laboratoy findings of patients before and after herpes virus infection treatment.

The result of serum antibodies of different viruses included CMV, EBV and HIV test are shown in Table 1. On April 16th, 2002, treatment with intravenous ganciclovir and 80,000U of interferon α sublingual every 8 h was commenced. Five days later her vital signs had improved with a respiratory rate of 36, pulse of 88 and weigh of 42 kg. One month after ganciclovir was commenced the lymphocytes count had risen to 3,148/*mm**^3^* and CD4+ T cells count was 322 cells/*mm**^3^* (10.2%) and CD8+ T cells count was 228 cells/*mm**^3^* (Fig. 2:A-C). She was treated with ganciclovir for 5 months and INFα for 8 months. In June 2003, her weight was 60 kg, respiratory rate 20, pulse 80 and her chest X-ray had only minimum residual lesions in the lower lung lobes (Fig. 1B). On October 14^th^, 2010 the patient was asymptomatic and her lymphocytes count was 1,988/*mm**^3^*, CD4+ T cells were 503/*mm**^3^* (25.3%), with 684 CD8+ T cells/*mm**^3^*. Also her CD4+/CD8+ T cells ratio was normal (Fig. 2: A-D).

### Patient 2:

A 76 year-old man with a history of arterial hypertension, atherosclerotic coronary disease and Kaposi's sarcoma ten years before, treated with chemotherapy, which was suspended in November 2006 because of recurrent episodes of fever, chills, anorexia and adynamia, diarrhea or constipation, sweating, a little coughing and headache. On June 7th, 2007 he had one of these episodes and fissures in his lips, tender bilateral cervical lymph nodes of 1.5 cm, Kaposi's sarcoma lesions in the legs, in the upper limbs, trunk and ear. His total lymphocytes count was 192 cells/*mm**^3^*, with 10 CD4+ T cells/*mm**^3^* (5.2%) and 137 CD8+ T cells/*mm**^3^* (Table 1 and Figure 2:A-C). The PCR for IS6110 sequence of *Mycobacterium tuberculosis * in urine and sputum were positive but ZN smear of gastric juice and sputum were negative and the mycobacteria culture of gastric juice were both negative and chest X-ray had not infiltrates. He had IgG antibodies to CMV and EBV (Table 1). On June 8^th^, he started taking valganciclovir for 3 months and clinical symptoms improved. The CD4+ T cells count and percentage, the CD8+ T cells count and the CD4+ T cells/CD8+ T cells of them ratio improvement are shown in Table 1 and Figure 2: A-D. In December 2007, he had again the adynamia, anorexia, vomitus, diarrhea, fever, shills, sweating, dyspnea and orthopnea. At the physical examination, he had oral candida lesions and a supraclavicular left lymph node of 1.5 cm, his skin lesions of Kaposi´s sarcoma have improved. His chest X-ray and chest CT showed little infiltrates in lower lung lobes and bilateral pleural effusion which were hemorrhagic. He started antituberculosis treatment on December 10th, with clinical improvement. On April 10^th^, 2008, he had 2,590 lymphocytes/*mm**^3^*, 811 CD4+ T cells/*mm**^3^* (31.3%) and 1,780 CD8+ T cells/*mm**^3^* (Fig. 2:A-C). In September 2009 the patient died of urinary sepsis.

### Patient 3:

A 44 year-old woman, a lawyer, was admitted to a clinic on August 21th, 2007 with a history of headaches over the past 10 months, with increased intensity for the last month and associated with mental organic and meningeal syndrome. She had focal neurologic alteration of the VI and VII left cranial nerves and left hemiparesis. She had suffered from recurrent oral ulcers for the last 6 years and appendectomy for the last 2 years. She related occupational stress in the last year, osteomyalgia and arthralgia with laboratory tests for SLE diagnosis negative. In a hemogram 8 months before, she had lymphocytopenia of 1,000cells/*mm**^3^*. At admission, the spinal fluid had glucose 11 mg/dl, proteins 95.9 mg/dl, leucocytes 0, erythrocytes 48, ZN smear and ink stain negative, fungus and mycobacteria culture negative. The PCR for IS6110 sequence of *M. tuberculosis* in cerebrospinal fluid was positive. Her peripheral blood lymphocytes count was 216/*mm**^3^*, with 25 CD4+ T cells/*mm**^3^* (11.6%) and 13 CD8+ T cells/*mm**^3^* (Table 1 and Fig. 2: A-C.) She had anti CMV IgG, EBV anti-capsid IgG and IFI to PP65 antigen of CMV positive, anti HTLVI-II negative and anti-HIV negative (Table 1). The brain CT and nuclear magnetic resonance were normal. The serum complement and DNA antibodies were negative. In August 21th, she started ganciclovir, dexametazone and 4 antituberculosis drugs with very good response. She was discharged 13 days after, with treatment with valganciclovir for 3 months and antituberculosis drugs for 9 months. On October 24th, 2007, she had not neurologic sequels. The CD4+ T cells count and percentage; CD8+ T cell count and CD4+ T cells/CD8+ T cells ratio improvement after the commencement of the treatment are shown in Table 1 and Fig. 2: A-D.

### Patient 4:

A 93 year-old man, a merchant, with antecedents of Parkinson's disease, cholecystectomy, choledocholithiasis and intestinal obstruction by brides and pneumonia in 2007, was admitted to the Intensive Care Unit on April 2^th^, 2009 with ten days history of fever, coughing, anorexia and weight loss. His vital signs were: respiratory rate of 30, pulse of 100 and blood pressure of 60/30 *mm*Hg. His chest X-ray showed alveolar infiltrates in both lower lung lobes. He did not respond to piperacilin-tazobactan treatment. His peripheral blood lymphocytes count was 500 cells/*mm**^3^*. A flow cytometry of peripheral blood showed 236 lymphocytes/*mm**^3^* with 68 CD4+ T cells/*mm**^3^* (28.8%) and 59 CD8+ T cells/*mm**^3^* (Table 1 and Figure 2:A-C). Serum creatinine 3.6mg/dl. On April 9^th^ he had *P. jiroveci* in a sputum and serum IgG antibodies to CMV and EBV (Table 1). On April 14^th^, he did start clindamycin, primaquine, prednisone and ganciclovir. On April 23^th^, after 9 days on this treatment, his lymphocytes raised to 874 cells/*mm**^3^* and his CD4+ T cells count rise to 495cells/*mm**^3^* (56.6%) and CD8+ T cell count 204 cells/*mm**^3^*. The evolution of other immunology parameters after the treatment is shown in the Table 1 and Fig. 2: A-D.

### Patient 5:

A 74 year-old woman with history of osteoporosis, gastric cancer 30 years before, cigarette smoke exposure, chronic obstructive pulmonary disease with spontaneous pneumothorax 8 months before, was attended by *Staphylococcus aureus* methicillin sensitive bacteremia and pneumonia. She was treated since April 1st, 2010 with piperacillin-tazobactam and mechanical ventilation support. The hemograms of April 1st and 9^th^ showed lymphocytopenia of 610 *mm**^3^* and 450 *mm**^3^* respectively. A chest CT showed findings of pulmonary thromboembolism, alveolar infiltrates in the left lung and interstitial fibrotic lesions, bulla, bilateral pleural effusion and right cardiomegaly (Fig. 1 C). On April 11^th^, she had 109 CD4+ T cells/*mm**^3^* (17.9%) and 43 CD8+ T cells/*mm**^3^* (Fig. 2: A-C). On April 9^th^ she was treated with trimethoprin sulfamethoxazole for S*tenotrophomonas maltophlia* nosocomial pneumonia and empirical for *P. jiroveci pneumonia.* Predisone plus ganciclovir were added. The results of antibodies to different virus are in Table 1. On April 27^th^, her lymphocytes count increased to 730/*mm**^3^*. On April 30^th^, she could be removed from mechanical ventilatory support. She was treated with valganciclovir for 3 months and voriconazole for 4 months, by repeated isolation of *Aspergillus fumigatus* in bronchial secretions without a lung biopsy because of the high risk of the procedure. In August she had an improvement in her immunology alterations which are shown in Table 1 and Figure 2: A-D. She also improved pulmonary infiltrates (Fig. 1: D).

### Patient 6:

A 62 year-old professor was diagnosed on November 7th, 2010 with coughing, adynamia, fatigue and progressive dyspnea for four months before. He had antecedents of amigdalectomy at 19 years old, hepatitis at 16 years old, appendectomy at 59 years old, red blood cells transfusion during diverticulitis with peritonitis in January 2006 and chronic renal diseases treated with hemodialysis until May 2007. Also he had a history of oral ulcers on two occasions in 2008 and urinary infection in January 2009. At the moment of medical examination, his chest X-ray showed diffuse bilateral interstitial infiltrates (Fig. 1E). The chest CT had reticular infiltrates greater in the right upper lung lobule, bronchiectasis, irregular interstitial subpleural thickening and ground-glass opacities superimposed. Previously, he had tuberculin test on 11 mm but the ZN smear mycobacterial and fungus culture of BAL and three ZN sputum were negative.

On November 17th, 2009, the lymphocytes count was 681/*mm**^3^*, CD4+ T cells were 279 / *mm**^3^* (40.9%) and CD8+ T cells were 110/*mm**^3 ^*(Table 1 and Fig. 2: A-C). He had IgG and IgM anti CMV negative but CMV PP65 antigen positive Table 1. On November 22^th^, 2009 a induced sputum calcofluor stain showed structures compatible with *P. jiroveci*. On November 23^rd^, he was treated with prednisone and trimethoprin-sulfamethoxazole for 3 days followed by clindamycin and primaquine for 18 days. On November 26^th^, he started ganciclovir for 3 months. On December 17^th^ the fever, cough, dyspnea, fatigue and weight loss had improved. On December 17^th^, 2009 the lymphocytes count was 957/*mm**^3,^* CD4+ T cells were 318/*mm**^3^* (33.2%) and CD8+ T cells were 182/*mm**^3^*. The improvement of different immunology alterations, after gancicolvir treatment, can be seen in Table 1 and Fig.2:A-D. The radiology pulmonary infiltrates improvement can be seen in the Fig.1: F.

## Discussion

This is the first report of HIV negative patients with lymphocytopenia and CD4 lymphocytopenia with clinical immunodeficiency who did improve their T CD4 count, their lymphocytes count and their illness with anti-human herpes virus treatment. All patients had serologic evidence of chronic CMV and EBV infections with positive antibodies IgG against both viruses except patient number 6, who had never developed IgG, neither IgM anti CMV although he had had blood transfusions around 3 years before and his CMV PP65 Ag was positive. All of them, except patient number 1 and patient number 5, whose CMV PP65 antigen was not carried out, had evidence of CMV virus active replication documented by this test positive in peripheral blood. These six patients had no focal clinical symptoms of disease by these viruses, although the first patient had EBV DNA in pulmonary biopsy and her chest X-ray interstitial infiltrates did clear up with ganciclovir treatment. The six patients had not evidence of acute infections because CMV and EBV antibodies IgM were negative in all of them except patient 1, who did not have the test. In all six patients the lymphocytes count and CD4+ T cells count, CD4+ T percentage, CD8+ T cells count and CD4 T cells/CD8 T ratio cell improved in a short time with the specific herpes virus treatment with ganciclovir or valganciclovir (Fig. 2: A-D). 

All six patients also had treatment for opportunistic infections such as TB or *P. jiroveci *pneumonia, but patient number 1 improved her symptoms, her lymphocytes count and CD4+ T cells count only when ganciclovir was given 17 days after she had finished trimethoprin sulfamethoxazole for *P. jiroveci* pneumonia with no improvement (Table 1 and Fig. 2: A and B) and patient number 2 had TB but the TB therapy was given only after his CD4+ lymphocytes had risen in response to anti-herpes virus treatment (Table 1 and Fig. 2: A and B).

The antiviral treatment was done to the other 4 patients along with the specific treatment for the opportunistic infections: extrapulmonary TB, (patient number 3) and *P. jiroveci* (patients 4, 5 and 6). It is possible that these opportunistic infections can cause CD4+ T lymphocytopenia. However, this effect reported with TB^3^ has not been as severe as it was in our patients who additionally had a non-severe negative bacilli TB, probed only by positive PCR for mycobacteria DNA.

CMV and other human herpes viruses are ubiquitous worldwide, especially in developing countries. After the viral infection is acquired, they are never completely eliminated. CMV can infect various cell types such monocytes, fibroblasts, endothelial cells, neutrophils, smooth muscle cells, neurons, epithelial cells and hepatocytes^4^ ^,^ ^5^. CMV establishes latency in monocytes and hematopoietic progenitor cells of myeloid lineage of bone marrow and recurrent reactivation occurs throughout life^4^ ^,^ ^5^.

EBV and CMV infections produce infectious mononucleosis after primary infection. Infectious mononucleosis has been described as a cause of transient CD4 T lymphocytopenia which improves with the patient's disease recovery^1^. Such lymphocytopenia has not been found in CMV or EBV asymptomatic, seropositive people, as was described before. On the other hand, severe lymphocytopenia and CD4+ T lymphocytopenia like that found in these six patients and associated with clinical immunodeficiency with opportunistic infections has not been reported in association with chronic herpes virus infections.

CMV has numerous strategies to evade the host immune surveillance. CMV decreases the cell surface expression of major histocompatibility complex molecules^4^ ^,^ ^5^. CMV and EBV both produce a homologue virokine to human IL-10, which affects T cell proliferation and dendritic cell functions. CMV induces apoptosis in dendritic cells, lymphocytes and neurons^4^ ^,^ ^5^. Also apoptosis was seen in tumoral lymphocytes in the liver of mice infected with mutant, non-disseminating CMV in their skin. A kind of virokine produced by CMV was proposed as a trigger of this effect^6^. Productive CMV infection in mesenchymal bone marrow stem cells changes the cell surface molecule expression, cytokines production and cell-cell interactions which affect cells differentiation and proliferation of bone marrow cells^7^. We think that these CMV immune effects could in chronic infected patients with active viral replication contribute to the decrease in the T cell counts in the peripheral blood of our patients and to the production of the clinical immunodeficiency.

The most known pathologies caused by CMV are seen in severely immunocompromised individuals or congenital infections but recently active CMV infection has been associated with severe diseases in immunocompetent hosts: gastrointestinal, neurologic and thrombotic diseases resembling lupus anticoagulant syndrome^8^. Seropositivity or viral antigens or DNA of CMV, EBV or herpes virus 6 were found in an increased number of patients with autoimmune diseases. CMV seropositivity is also associated with aging of the immune system characterized by dramatic reduction of naïve and early memory CD8 T cells and accumulation of CD8+ CD28- and CD27- senescent effector T cells^4^.

The six cases reported in this paper show a new clinical manifestation associated with chronic infection by human herpes CMV and EBV: profound lymphocytopenia and CD4+ T lymphocytopenia with clinical immunodeficiency and opportunistic infections in persons without immunodeficiency history and no other cause of it. This is the first paper showing the improvement of profound lymphocytopenia and CD4+ T lymphocytopenia with treatment of chronic infections by human herpes virus. Thus, we propose that chronic CMV and/or EBV and/or human herpes virus 6 (HHV-6) that infect TCD4 cells and TCD8 cells and that can respond to ganciclovir treatment (HHV-6 was not investigated in these patients), should be kept in mind during the differential diagnosis of idiopathic CD4 lymphocytopenia (ICL). This is a pathology defined by the Centers for Disease Control and Prevention (CDC) as patients with numbers of circulating CD4 T lymphocytes < 300 cells/*mm**^3^* or < 20% of total T cells on a minimum of two separate measurements at least 6 weeks apart, with no laboratory evidence of infection with human HIV-1 or HIV-2 and with the absence of any defined immunodeficiency or therapy associated with depressed levels of CD4 T cells.. Some cellular alterations have been reported in cases of ICL: a 50% reduction of p56 (Lck) kinase activity in the T cells in one patient^9^, missense mutations in RAG1 resulting in reduced RAG activity in another patient^10^, abnormal expansion of TCR alpha beta with reduced export of mature T cells from the thymus, in one adolescent^11^ and a defect in CXCR 4 expression on the surface of CD4+ T cells with an intracellular accumulation of CXCR4 and CXCL12 in 6 patients^12^. However the majority of ICL cases remain without known molecular alteration. In some of the reported cases, immune response alterations have been found, which are similar to the immune effects of human herpes virus, especially CMV. Some examples are: a decreased numbers of naive T cells (CD54RA+), a relative expansion of memory T cells (CD45 RO+) and a higher percentage of T regulatory cells, increased CD4 T-cell activation and turnover, increased apoptosis of CD4 lymphocytes, reduced lymphocytes proliferation response and restricted oligoclonal T cells antigen repertoire, disturbance in the signaling of T cells antigen receptor and deficient IL2 production^1^,^13^. These similarities between the ICL and immune CMV effects and the occurrence of CMV and EBV encephalitis, CMV digestive compromise and retinitis in some reported ICL cases^1^ ^,^ ^14^ ^,^ ^15^ lead us to propose that some of the ICL cases reported before, could be really secondary to CMV or EBV or other ganciclovir sensible chronic human herpes virus.In summary, the rapid response of lymphocytopenia and CD4+ T lymphocytopenia in all 6 patients chronically infected by human herpes virus CMV plus EBV, with specific treatment of these viruses, is good evidence of a new associated clinical manifestation to chronic herpes virus infection by CMV with active replication or reinfection, EBV or herpes 6. This association must be considered in the differential diagnosis of CD4+ T lymphocytopenia, in HIV negative individuals. To think, these new observation could help to identify patients susceptible to treatment and to heal them. Also this report allows for deeper research about how and which of chronic human herpes virus infection are important in the developing disease.
